# Clinical and biochemical outcomes of Sodium-Glucose CoTransporter-2 (SGLT2) Inhibitors in Type-2 Diabetes Mellitus Patients as a fourth oral anti diabetic medicine

**DOI:** 10.12669/pjms.38.5.5034

**Published:** 2022

**Authors:** Muhammad Saleem, Sajjad Ali Khan, Zafar Aleem Suchal, Nanik Ram

**Affiliations:** 1 Muhammad Saleem, FCPS, Fellow- Section of Endocrinology, Department of medicine, Aga Khan University Hospital, Karachi, Pakistan; 2 Sajjad Ali Khan, FCPS, Fellow- Section of Endocrinology and Department of Medicine, Aga Khan University Hospital, Karachi, Pakistan; 3 Zafar Aleem Suchal, Medical Student, Aga Khan University Hospital, Karachi, Pakistan; 4 Nanik Ram, FCPS, Assistant Professor in Section of Endocrinology and Department of Medicine, Aga Khan University Hospital, Karachi, Pakistan

**Keywords:** SGLT2 Inhibitors, T2DM, Weight loss

## Abstract

**Objectives:**

To evaluate the clinical and biochemical effects of (SGLT2) inhibitors as a fourth oral anti-diabetic drug in patients with Type-2 diabetes mellitus (T2DM).

**Methods:**

In a tertiary hospital in Karachi, Pakistan, a retrospective assessment of patient medical records was conducted from January 1, 2017 to December 31, 2020.A total of one hundred patients (mean age [Standard Deviation]: 53.8 [9.63] years) with poorly controlled T2DM were included. Data was collected before the SGLT-2 inhibitor was added, as well as three and six months after the medicine was started. Weight, BMI, blood pressure (BP), HbA1c, SGPT, and Creatinine were measured at the start and during the study

**Results:**

There was a significant reduction in HbA1c (p-value < 0.001) with a mean reduction (MR) of 0.81+1.02% at three months and 1.07+1.11% at six months. A mean weight reduction (p-value < 0.001) of 1.83+2.32 kg at three months and 4.02+6.04 kg at 6 months, respectively, was recorded. A mean BMI reduction of 0.69+0.95 kgm-2 at three months and 2.13+3.41 kgm-2 at six months of follow up, respectively were recorded. A systolic blood pressure (SBP) also showed a significant reduction (p-value < 0.05) with a MR of 5.9+15.76 mmHg at three months and 6.37+18.33 mmHg at 6 months, respectively. Non-significant variation in creatinine and SGPT was also noted.

**Conclusions:**

SGLT-2 is an effective oral anti-diabetic medicine that can help individuals with diabetes who are currently using glucose-lowering oral anti-diabetic medications. These medications can help diabetic patients stick to their regimen.

## INTRODUCTION

Diabetes mellitus is a group of related metabolic disorders characterized by hyperglycemia ensuing from defects in insulin production, insulin action, or both. Persistent hyperglycemia is linked to long-term injury, dysfunction, and failure of multiple organs, including the eyes, kidneys, nerves, heart, and blood vessels. Diabetes may be classified into type one diabetes mellitus (due to β-cell destruction, typically resulting in absolute hormone deficiency), Type-2 diabetes mellitus (due to a progressive hormone humoral defect on the background of hormone resistance), Gestational DM (GDM) (during pregnancy, diabetes is diagnosed) and Specific forms of diabetes because of alternative causes.^1^

According to the International Diabetes Federation, around 415 million individuals worldwide were diagnosed with diabetes in 2015, with that figure anticipated to rise to 640 million by 2040.^2^ A meta-analysis conducted on the Pakistani population by Sohail et al., the prevalence of Type-2 diabetes in Pakistan is 14.6%. Microvascular and macrovascular problems are the two types of chronic diabetic complications, with the former having a far higher prevalence than the latter.^3^ Microvascular complications include neuropathy, nephropathy, and retinopathy, whereas macro vascular complications consist of cardiovascular disease, stroke, and PAD. Diabetic foot syndrome is characterized as a foot ulcer accompanied by neuropathy, peripheral artery disease, and infection, and it is a leading cause of lower limb amputation.^4^

An antidiabetic drug from no less than eight pharmacologic classes is chosen for the treatment of Type-2 diabetes mellitus. Agents that boost insulin secretion, improve insulin action, and delay glucose absorption fall within this category.^5^ Patients with Type-2 diabetes commonly begin treatment with oral medicines such as metformin or sulfonylurea as well as life style adjustments, and later move to combination therapy. The majority of patients require three or more medications, as well as insulin. There are many options available for additional therapy including dipeptidyl peptidase-4 Inhibitors, thiazolidinedione, intermediate- and long-acting insulins and Glucagon-like peptide-1 receptor agonists. Despite the fact that the American Diabetes Association supports metformin as first-line therapy, it does not provide specific recommendations for second- and third-line medicines, instead summarizing clinical facts and options for each therapeutic decision.^6^

Sodium/glucose cotransporter-2 inhibitors (SGLT2i) function independently of insulin, reduce blood glucose by inhibiting its reabsorption in proximal tubules and by promoting urinary glucose excretion, without inducing hypoglycemia or weight gain. The Objective of our study was to evaluate the clinical and biochemical effects of (SGLT2) inhibitors as a fourth oral anti-diabetic drug in patients with Type-2 diabetes mellitus (T2DM).

## METHODS

This was a retrospective, real world, single center observational study carried out on patients of Aga Khan University Hospital (AKUH), a tertiary hospital in Karachi, Sindh, Pakistan between January 2017 and December 2020. The study included patients of either sex having Type-2 diabetes. Inclusion criteria was defined as patients with age more than 16, already being treated with three oral anti-diabetics (Metformin, DPP-4 inhibitors and Sulphonylureas), Glycated Hemoglobin (HBA1C) of more than 7% , and with an estimated Glomerular Filtration Rate (eGFR) more than 45. All Patients that met these criteria were added SGLT-2 inhibitor namely empagliflozin or dapagliflozin as a fourth anti-diabetic drug to bring Fasting Blood Glucose (FBS) to an acceptable level of 90 – 120 mg/dL and to lower the HbA1c. All the patients were enrolled through convenience sampling. Patients with Type-1 diabetes mellitus were excluded from our study. Additionally, Type-2 diabetics who were also being treated with insulin or GLP-1 agonist were excluded from our study. The parameters assessed at baseline before the addition of SGLT-2 inhibitor included physical parameters such as Systolic Blood Pressure (SBP), Diastolic Blood Pressure (DBP), Weight and Body Mass Index (BMI) and biochemical parameters such as Glycated Hemoglobin (HbA1c), Serum Glutamic Pyruvic Transaminase (SGPT) and Creatinine (Cr).

### Endpoint Assessment:

All the parameters assessed at baseline before the addition of SGLT-2 inhibitor were also assessed at 3-month interval and at 6-month interval. These included clinical parameters recorded at each clinical visit like Weight recoded by a weighing balance, BMI calculated in the clinic, SBP and DBP by a digital sphygmomanometer. Biochemical markers recorded included HbA1c, SGPT and Cr.

### Statistical Methods:

All statistical analysis was performed using Statistical package for social science SPSS (Release 19.0, standard version, copyright © SPSS; 1989-02) and MS-Excel 2016. Comparison between baseline and follow up value of various parameters was done using paired t test. Continuous variables were presented as mean (standard deviation [SD]) with 95% CI as applicable and categorical data were presented as proportions. All P values were 2-sided and were considered significant if P < .05. The comparison between baseline and follow up values was done at 3 months and 6 months after the addition of SGLT-2 inhibitor.

## RESULTS

A total of one hundred patients were taken for this study with 49 females and 51 males. All the parameters required for analysis were recorded before addition of SGLT-2 inhibitor. The baseline parameters have been given in Table-I. The outcome in terms of physical parameters (Weight, BMI and Blood Pressure) and in terms of biochemical parameters (HbA1c) was recorded at two intervals 3 and 6 months after the addition of SGLT-2 inhibitor. The mean improvement in these parameters has been summarized in Table-II. The mean HbA1c showed a significant decrease after the start of therapy with the mean improvement after 3 months being of 0.81 and after 6 months of 1.07. Both values being statistically significant (P <.001).There has been a statistically significant (P *<*0.001) decrease in weight with a mean decrease of 1.83 kg at 3 and 4.02 kg at six months.The BMI of patients has decreased with a decrease of 0.69 kgm^-2^ at three months and 2.13 kgm^-2^ at 6 months, both being statistically significant (P < .001).

**Table I:** Baseline Demographics of the study group.

Variable	Baseline Mean (SD)*
Age (years)	53.80 (9.63)
Male	51
Female	49
Duration of Diabetes (years)	9.59 (5.64)
HbA1c* (%)	8.23 (1.09)
Weight (kg)	81.78 (13.69)
BMI* (kgm^-2^)	31.35 (5.43)
SBP* (mmHg)	139 (19)
DBP* (mmHg)	75 (11)
Creatinine (mg/dl)	0.83 (0.25)
SGPT* (IU/L)	34.71(8.34)

HbA1c: Glycated Hemoglobin, BMI: Body Mass Index, SBP: Systolic Blood Pressure, DBP: Diastolic Blood Pressure, SD: Standard Deviation, SGPT: Serum Glutamic Pyruvic Transaminase

**Table II:** Improvement in Clinical and biochemical Parameters.

	Improvement at 3 months				Improvement at 6 months			

Variables	Mean Improvement	SD*	95% CI* (LCL, UCL)	P-Value	Mean Improvement	SD*	95% CI* (LCL, UCL)	P-Value
Weight (kg)	1.83	2.32	1.30-2.35	< 0.001	4.02	6.04	2.69-5.35	< 0.001
BMI* (kgm^-2^)	0.69	0.95	0.48-0.90	<0.001	2.13	3.41	1.03-2.44	<0.001
SBP* (mmHg)	5.9	15.76	2.20-9.61	0.002	6.37	18.33	2.18-10.56	0.003
DBP* (mmHg)	2.65	16.08	-1.08-6.37	0.161	2.60	14.67	-0.704-5.91	0.121
HbA1c* (%)	0.81	1.02	0.60-1.02	< 0.001	1.07	1.11	0.83-1.23	< 0.001
Creatinine	0.85	0.26	0.79-0.92	< 0.001	0.82	0.22	0.77-0.87	< 0.001
SGPT	29.74	13.42	25.61-33.87	< 0.001	31.93	16.22	27.50-36.35	< 0.001

HbA1c: Glycated Hemoglobin, BMI: Body Mass Index, SBP: Systolic Blood Pressure, DBP: Diastolic Blood Pressure, SD: Standard Deviation, CI: Confidence Interval,

* SGPT: Serum Glutamic Pyruvic Transaminase

The SBP of the patients has shown a statistically significant decrease with the mean decrease being 5.9 mmHg at 3 months (P = .002) and 6.37 mmHg at 6 months (P = .003).The DBP of patients has also decrease however the decrease has not been statistically significant with P-value > .05.The renal and hepatic function over the 6 months’ period were assessed using serum creatinine and SGPT of the patient. The mean of both these variables remained within the normal range over the 6 months follow up with the results being statistically significant (P < .001) as shown in Table-III.

**Table III:** Creatinine and SGPT of the patients over the study course.

	Creatinine				SGPT*			

	Mean	SD	95% CI (LCL, UCL)	P-value	Mean	SD	95% CI (LCL, UCL)	P-value
Baseline	0.83	0.25	0.78-0.88	< 0.001	34.71	24.34	29.15-40.27	< 0.001
3 months after adding SGLT-2 inhibitor	0.85	0.26	0.79-0.92	< 0.001	29.74	13.42	25.61-33.87	< 0.001
6 months after adding SGLT-2 inhibitor	0.82	0.22	0.77-0.87	< 0.001	31.93	16.22	27.50-36.35	< 0.001

* SGPT: Serum Glutamic Pyruvic Transaminase

## DISCUSSION

In this study, we observed the effect of addition of SGLT-2 inhibitors, as a fourth anti diabetic drug, on various clinical and biochemical parameters including Weight, BMI, SBP, DBP, HbA1c, SGPT and Creatinine at 3months and 6 months interval for 100 diabetic patients already undergoing treatment with three oral anti diabetic drugs. Studies of similar have been conducted on different populations however local data is still very scarce particularly regarding the addition of SGLT-2 inhibitors as a fourth anti diabetic drug. Furthermore, impediments to adhering to injectable Insulin therapy, such as the patient’s dread of injectable and the discomfort they cause, as well as refusal and noncompliance, can result in poor glycemic control and complications.^7^,^8^ This study contributes to the understanding of the advantages of using SGLT-2 inhibitors as a fourth oral anti-diabetic agent instead of injectable insulin.

Systolic Blood Pressure, Diastolic Blood Pressure, body weight, and HbA1c levels have all improved significantly after taking SGLT-2 inhibitors in previous research.^9^-^11^ An expert analysis studying similar population also showed the medications to have a beneficial effect on these parameters.^12^ Our results were in coherence with previous studies showing a significant improvement in HbA1c, Weight, BMI, SBP, SGPT and Creatinine with addition of SGLT-2 inhibitors. Meta-analysis, conducted by Storgaard H et al., of 34 RCT’s showed a reduction of HbA1c levels by use of SGLT-2 inhibitors.^13^ Our study has shown similar results with HbA1c levels being significantly reduced at 3 months and 6 months follow up with mean improvement of 0.81% and 1.07% respectively.

Reduction in weight was observed in another meta-analysis when SGLT-2 inhibitor was added to Metformin as dual therapy.^14^ In our analysis significant reduction in weight was observed at 3-month intervals with mean reduction of 1.83 kg and at 6-month interval with mean reduction of 4.02 kg.

BMI reduction was reported after use of empagliflozin a SGLT-2 inhibitors by several studies including a meta-analysis conducted by Chewcharat A et al. on diabetic patients having undergone kidney transplant.^15^ Our research found similar results, with a substantial mean reduction in BMI of 0.69 kgm^-2^ and 2.13 kgm^-2^ at three and six months’ intervals. In previous studies it was shown that addition of SGLT-2 inhibitors with Metformin as a dual therapy, showed significant reduction in SBP as well as DBP.^13^ In our study the SBP showed significant mean reduction of 5.9 mmHg at 3 months and 6.37 mmHg at 6 months. The mean reduction in DBP in our study was observed to not be significant. Future studies on the Pakistani population will be required to track the DBP trend with the inclusion of SGLT-2 inhibitors.

Although earlier research has linked the use of SGLT-2 inhibitors to an increase in Creatinine,^13^,^16^ the mean creatinine level in our sample exhibited little fluctuation. level from 0.83 at the start of treatment to 0.85 at 3 months’ interval and 0.82 at 6 months’ interval. Improvements in liver function have been documented in earlier trials, which have been linked to the metabolic effects of SGLT-2 inhibitors. The mean SGPT levels varied from 34.71 at baseline to 29.74 after three months and 31.93 after six months, demonstrating no substantial decline in liver function.^17^

### Limitations of the study:

This is a modest, single-center study with a sample size of 100 patients. Future multicenter trials with a larger sample size and conducted over a longer time period will be required to assess the long-term safety profile of SGLT-2 inhibitors. This discovery paves the way for more research into the health benefits of SGLT-2 inhibitors in the general population.

## CONCLUSION

Our research has demonstrated that SGLT-2 inhibitors is a safe and effective oral anti-diabetic therapy that can help individuals with diabetes who are currently using glucose-lowering oral anti-diabetic medications. These medications can be used as an alternative to injectable insulin for people who do not want to use it, and they can help diabetic patients stick to their regimen.

### Authors Contribution:

**MS:** Conceived, designed, and did statistical analysis.

**SAK:** Did data collection and manuscript writing.

**ZAS:** did data analysis, the editing of the manuscript.

**NR:** Did a review and final approval of the manuscript.
